# Adjusted versus Targeted Fortification in Extremely Low Birth Weight Preterm Infants: Fortin Study—A Randomized Clinical Trial

**DOI:** 10.3390/nu16172904

**Published:** 2024-08-30

**Authors:** Maria Sanchez-Holgado, Miguel Saenz de Pipaon, Maria Concepcion Jimenez, Gema Crespo Sanchez, Marta Molero-Luis, Maria Teresa Montes, Cristina Segovia, Itsaso Losantos-García, María Jimenez-Gonzalez, Esperanza Escribano, Marta Cabrera-Lafuente

**Affiliations:** 1 Neonatology Hospital La Paz Institute for Health Research, IdiPaz (Universidad Autonoma de Madrid), 28046 Madrid, Spain; msholgado@salud.madrid.org (M.S.-H.);; 2 Department of Neonatology, La Paz University Hospital, 28046 Madrid, Spain; 3 Laboratory of Gastroenterology and Trace Elements, Department of Laboratory Medicine, La Paz University Hospital, 28046 Madrid, Spain; mariagema.crespo@salud.madrid.org (G.C.S.);; 4 Hospital La Paz Institute for Health Research-IdIPAZ, La Paz University Hospital, 28046 Madrid, Spain; 5 Clinical trials Unit, La Paz University Hospital, 28046 Madrid, Spain

**Keywords:** preterm infants, neonatal nutrition, growth, human milk, human milk fortification, blood urea nitrogen, individualized fortification

## Abstract

Fortified human milk is the first choice for preterm infants. Although individualized fortification is recommended, the optimal method for this population remains uncertain. We conducted a comparative study assessing the growth effects of adjusted (AF) and targeted fortification (TF) in extremely low birth weight (ELBW) infants. This single-center, randomized, controlled clinical trial was conducted at a tertiary neonatal unit in Spain. Eligible participants were premature infants with a birthweight of <1000 g exclusively fed with human milk. A total of 38 patients were enrolled, 15 of them randomized to AF group and 23 to TF group. AF was based on blood urea nitrogen (BUN) concentration and TF on human milk analysis. The primary outcome was weight gain velocity (g/kg/day). No significant differences were found in weight gain velocity at 28 days, at 36 weeks of postmenstrual age, at discharge, nor during the intervention. Protein intake was significantly higher in the AF group (5.02 g/kg/day vs. 4.48 g/kg/day, *p* = 0.001). No differences were found in the lipid, carbohydrate, and energy intake; in the weight z score change between the different time points; nor in the length and head circumference growth. Both AF and TF are comparable methods of fortification and provide the appropriate growth rate in ELBW infants.

## 1. Introduction

Human milk (HM) is the preferred choice for preterm infants, and it is associated with multiple short- and long-term benefits [1,2,3,4,5,6,7]. When mother’s own milk (MOM) is not available, donor human milk (DHM) is the best alternative [8,9].

Despite its advantages, MOM or DHM do not meet the nutritional requirements of preterm infants, especially in terms of protein, calcium, and phosphorus. This is particularly true for extremely low birth weight (ELBW) infants due to their accelerated growth [1,10,11,12,13]. The goal of fortification is to increase the nutrient concentration of HM to the level required to optimize growth [8,14,15,16,17,18,19].

There are three approaches for fortifying HM for ELBW infants. Standard fortification (SF), the most widely used strategy, consists of adding a fixed amount of multicomponent fortifier per 100 mL of HM during the entire fortification period [1,16]. This method assumes a fixed and estimated HM composition with a protein content of 1.5 g/dL, without considering temporal or inter-individual variations [13]. However, this assumption may not be accurate [20], and this strategy has been shown to lead to suboptimal growth in ELBW infants [15,21,22,23,24].

Individualized fortification is emerging as a potential solution to mitigate the inherent variability in HM composition. However, the optimal fortification strategy remains uncertain [24,25]. It involves two different methods as follows: adjusted fortification (AF) based on blood urea nitrogen (BUN) concentration as a marker of protein nutrition [26,27,28,29] and targeted fortification (TF) based on the regular analysis of HM and fortification adjustment according to the actual macronutrient composition [30,31]. It allows tailor-made fortification to meet the required nutritional recommendations. Despite the theoretical advantages of this nutritional strategy, its implementation presents significant challenges [32,33,34,35,36,37,38].

The aim of this study is to compare the effects of AF and TF on growth and neonatal morbidity in ELBW preterm infants.

## 2. Materials and Methods

### 2.1. Study Design

A prospective, single-center, randomized, controlled, interventional study performed in a tertiary referral neonatal unit at La Paz University Hospital (Madrid, Spain). The study protocol was approved by the local ethics committee. Written informed parental consent was obtained. The trial was registered at ClinicalTrials.gov with identification number NCT04982133.

#### A Priori Research Hypothesis

Targeted fortification will improve postnatal weight gain and growth over an adjusted fortification.

### 2.2. Participants

Preterm infants with a birthweight <1000 g fed with MOM or DHM were eligible for the study when they reached an enteral feeding volume ≥100 mL/kg/day. Exclusion criteria included major congenital malformation, chromosomopathies, metabolic disease or gastrointestinal surgery.

#### Randomization

Patients whose parents signed the informed consent were randomized to a fortification method. Initially, a stratified randomization method was proposed, based on intrauterine growth restriction (IUGR), defined as a birth percentile <3 or <10 with abnormal prenatal Doppler. However, due to the low incidence of IUGR in the study population, the protocol was adjusted to a simple randomization. A list of unique randomization codes was followed and assigned consecutively to each study participant. Siblings from multiple births were randomized individually.

Once the patient was assigned to a study group, the intervention could not be blinded to the investigators or the attending physicians due to the nature of the nutritional prescription. However, only the investigators made changes to the fortification in order to avoid deviations in the intervention. The results of the HM analysis in AF group were blinded until the end of the study.

### 2.3. Nutritional Intervention

All infants initially received standard fortification with a multicomponent fortifier (PreNAN Human Milk Fortifier™, Nestlé, Vevey, Switzerland). This fortifier was administered at the recommended dosage of 1 g per 25 mL, providing an additional 0.7 g of fat, 1.42 g of protein, 1.3 g of carbohydrates, and 17.4 kcal per 100 mL of human milk. After this standard fortification, the infants were randomly assigned to one of the following two parallel treatment groups: (1) AF, where fortification was modified based on BUN concentration and (2) TF, where fortification was adjusted according to the macronutrient content of the human milk.

Nutritional intake was assessed twice a week. The intervention continued until 36 weeks of postmenstrual age (PMA) or until discharge, whichever came first. The study was also discontinued when DHM was replaced by formula due to hospital criteria (weight >1500 g or age >6 weeks). Parenteral and enteral feeding regimens were standardized by the internal feeding guidelines, which remained unchanged throughout the study period. The target volume for enteral nutrition was 150–180 mL/kg/day. MOM feeding was supplemented with DHM when necessary to achieve adequate enteral intake.

#### 2.3.1. Adjusted “Modified” Fortification

Adjustments were made by BUN concentration weekly [26,27]:

- BUN < 10 mg/dL: Protein fortification was increased by one level by adding a protein fortification module [oligopeptides (Clinical Nutrition, SA, Barcelona, Spain) 0.5 g/100 mL, 1 g/100 mL or 1.5 g/100 mL]. Protein fortification was progressively increased (0.5–1–1.5 g/100 mL) each week if BUN levels remained below 10 mg/dL.
- BUN 10–16 mg/dL: No nutritional changes were performed if growth was adequate, which was defined as no decrease in the weight z-score on the Fenton curves compared to the previous weekly weight, after the postnatal weight loss phase [39,40].
- BUN > 16 mg/dL: Protein fortification was decreased by one level if growth was adequate.
- If BUN > 10 mg/dL but growth was inadequate, which was defined as a decrease in the weight z-score on the Fenton curves, an insufficient intake of other macronutrients was inferred, and non-protein kilocalories were supplemented with lipids in the form of medium-chain triglycerides [MCT 1–2 mL/kg (Nutricion Medica, Madrid, Spain)] and glucose polymer powder [0.97 g carbohydrates/g (Fantomalt, Nutricia, The Netherlands) 2 g/100 mL].

**Figure 1:**
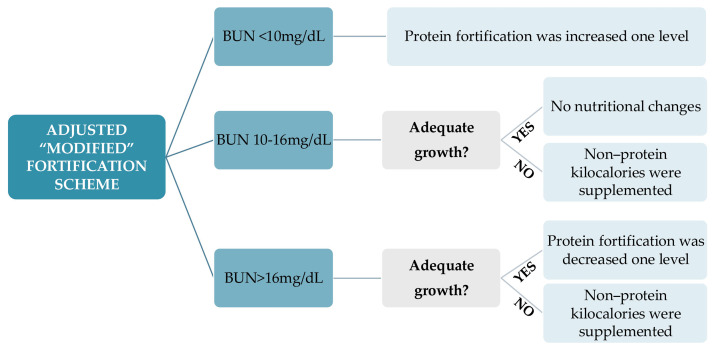
Adjusted “modified” fortification scheme. Adequate growth was defined as no decrease in weight z-score on Fenton curves compared to the weight measurement obtained during the previous weekly nutritional assessment.

Multicomponent fortification was never withdrawn, irrespective of the BUN value. The adjusted “modified” fortification scheme is illustrated in Figure 1.

#### 2.3.2. Targeted Fortification

Adjustments were made according to the twice-weekly macronutrient analysis of HM. Monocomponent modules (oligopeptides, MCT or glucose polymer powder) were added to reach the nutritional targets recommended by the European Society of Pediatric Gastroenterology, Hepatology, and Nutrition (ESPGHAN), in relation to enteral intake in preterm infants [41] as follows: protein 3.5–4.5 g/kg/day, carbohydrate 11.6–13.2 g/kg/day, lipids 4.8–6.6 g/kg/day, and energy 110–135 kcal/kg/day. The aim of these adjustments was to consistently target the upper end of the recommended range. These adjustments were based on the actual intake volume on the day of nutritional assessment. Initial fortification with the multicomponent fortifier was never withdrawn even though some macronutrients exceeded the nutritional recommendations.

The amount of additional fortification required to reach ESPGHAN targets was calculated for each macronutrient using a standardized study recipe sheet that took into account the actual macronutrient analyses of HM, volume intake, and actual weight (See Supplementary Materials).

### 2.4. Analysis of Human Milk

Macronutrient composition analysis of MOM or DHM was performed in both groups twice a week. The analysis focused on the predominant type of HM, defined as the HM type that represented more than 50% of the intake during the previous 3 days. The analysis was performed on an 8 mL aliquot obtained from a 24 h pool of fresh HM. If fresh milk was not available, frozen milk was used, after being thawed 24 h in a refrigerator at 4 °C, according to the recommendations for thawing HM [42]. All samples were analyzed using a milk analyzer based on near infrared (NIR) spectroscopy (MilkoScanTM Mars, FOSS Analytical A/S, Hillerød, Denmark). The HM composition of protein (true protein, g/100 mL), carbohydrate (lactose, g/100 mL), and fat (g/100 mL) were obtained, as well as a calculated value of kcal/100 mL. Regarding energy calculation, we utilized the following standard equation to determine the energy content of HM: we multiplied grams of protein by 4 kcal/g, grams of carbohydrates by 4 kcal/g, and grams of fats by 9 kcal/g, and summed these values to obtain the total kcal/dL.

Analysis of HM composition was integrated into the routine laboratory procedures. Laboratory professionals performed the equipment verification and control, and they own a quality accreditation (UNE-EN-ISO-15189 standard) [43].

### 2.5. Biochemical Analyses

In the AF group, analysis of the serum urea, calcium, phosphorus, and alkaline phosphatase in the venous blood was performed weekly. In the TF group, the same serum measurements were performed but only 28 days after the beginning of fortification, at 36 weeks PMA, and at discharge. Serum urea levels were determined using the urease method. An automated clinical analyzer (Siemens CH Analyzer, Siemens Healthineers, Erlangen, Germany) was used.

### 2.6. Outcomes

The primary outcome was weight gain velocity (g/kg/day) in the following periods: birth to 28 days after the initiation of fortification, birth to 36 weeks PMA, birth to discharge, and during the intervention. Weight was recorded daily to the nearest 5 g using an electronic scale (Kern MBC 15K2DM, Kern and Sohn, Balingen, Germany) or an incubator with adequate calibration (Babyleo TN500, Draeger, Lübeck, Germany).

Weight gain velocity was calculated according to Patel et al.’s formula [44] as follows:

[1000 × ln(Wn/W1)]/(Dn − D1)

where W1 is the weight at the start and Wn is the weight at the final day of the observation. D1 is the starting day and Dn is the final day of the observation period.

The research team performed all the anthropometric measurements.

The secondary outcomes were as follows:

1. Length and head circumference (HC) growth: Cranial–heel length was measured on a length board in supine position (Añó sayol, Barcelona, Spain), and HC was measured with a non-stretching tape to the nearest 0.5 cm.
2. Standard deviation score (SDS) differences: Based on Fenton graphs [41] for weight, length, and HC between the periods studied (SDS different time points—SDS birth).
3. Actual enteral macronutrient intake (g/kg/day and Kcal/kg/day).
4. Nutritional achievements: initiation of enteral nutrition (hours), exclusive enteral nutrition (150 mL/kg/day) (days), parenteral nutrition cessation (days), start of HM fortification (days), and volume of enteral feeding at the beginning of fortification (mL/kg/day).
5. Predominant HM type (>50% of intake during the study period): MOM or DHM.
6. Perinatal characteristics and neonatal morbidities: early-onset sepsis [45], intraventricular hemorrhage ≥ grade II [46], white matter injury [47], significant ductus arteriosus [48,49], necrotizing enterocolitis defined as Bell’s stage > 2 [50], retinopathy [51], moderate-severe bronchopulmonary dysplasia [52,53], cholestasis [54], late-onset sepsis [45], and death.
7. Neonatal intensive care unit (NICU) and hospital stay (days).

### 2.7. Sample Size and Statistical Analysis

Sample size was calculated based on the results of previous studies, where the reported weight gain was 19.9 ± 2.7 g/kg/day for TF [30] and 17.5 ± 3.2 g/kg/day for AF [26]. The calculation was performed assuming a one-sided test, based on the hypothesis that TF would improve postnatal weight gain and growth compared to AF. A test power of 80% was used, resulting in a sample size of 38 participants.

Qualitative data are described in absolute frequencies and percentages and quantitative data by mean and standard deviation or median and interquartile range, based on its distribution. The normality of the continuous variables was studied using the Kolmogorov–Smirnov test.

The association between variables was studied using the chi-squared test for categorical variable and Student’s *t*-test, as a parametric test, or the Mann–Whitney U test, as a non-parametric test, for continuous variables. To study the relationship between the quantitative variables, Pearson’s correlation or, its non-parametric equivalent, Spearman’s correlation were used. Generalized linear repeated measures models were used to assess the relationship between protein intake and urea values. For all statistical tests, 95% confidence intervals were calculated.

All statistical tests were considered bilateral and *p*-values less than 0.05 were considered significant. Data were analyzed with the SAS statistical software 9.4 (SAS Institute, Cary, NC, USA).

## 3. Results

**Figure 2:**
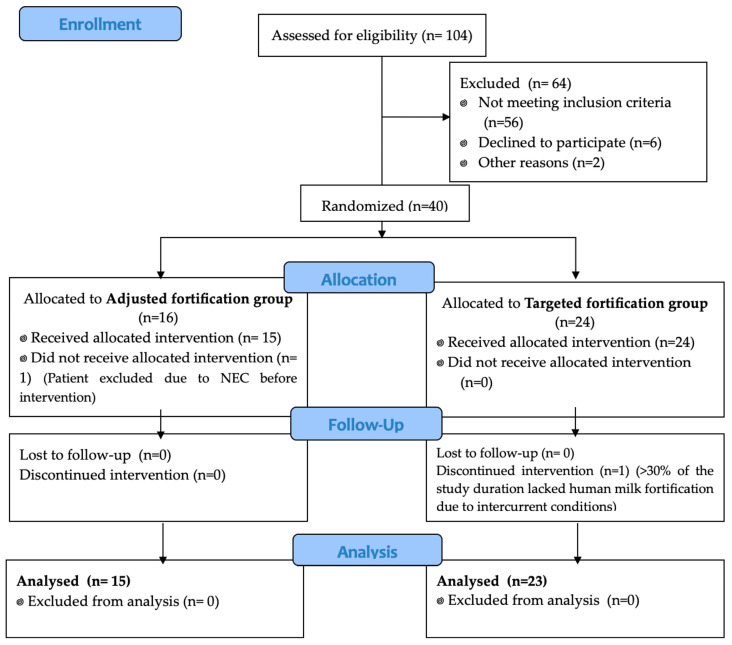
CONSORT flow chart.

A total of 38 patients were included from April 2021 to April 2023 (104 preterm infants < 1000 g were admitted to the NICU in this period; Figure 2), and 15 patients were randomized to the AF group while 23 were randomized to the TF group.

Mean (SD) gestational age was 27 weeks (2) and birth weight (interquartile range-IQR) was 835 g (710–894). Baseline general characteristics were comparable between both arms (Table 1). Other characteristics were as follows in the AF and TF group, respectively: complete lung maturation (two corticosteroid doses), 87% vs. 74%; incidence of chorioamnionitis, 13% vs. 35%; born by cesarean section, 87% vs. 74%.

The mean (SD) duration of the intervention was 50 (14) days in the AF group and 46 (16) days in the TF group (*p* = 0.40).

### 3.1. Nutritional Strategy

Enteral nutrition was started at 24 h in both groups (IQR AF 20–48 h and TF 20–30 h, *p* = 0.5), exclusive enteral nutrition was reached at 17 (IQR 13–26) days in the AF group and at 15 (IQR 12–25) days in the TF group (*p* = 0.48). No differences were found between the groups in terms of age (SD) at parenteral nutrition discontinuation [AF 17 (7) days vs. TF 16 (9) days, *p* = 0.91].

Fortification was initiated in the AF group at 15 (IQR 11–22) days and in the TF group at 14 (IQR 10–21) days (*p* = 0.67). There was no significant difference in the volume of enteral nutrition at which fortification was initiated [AF 146 mL/kg/day (IQR 121–152) vs. TF 145 mL/kg/day (IQR 123–155), *p* = 0.89]. The predominant type of feeding was MOM in both groups with a frequency of 73% in AF group and 52% in TF group (*p* = 0.192).

No differences were found in the HM composition between the groups in terms of protein, fat, lactose, and energy. Protein intake was significantly higher in AF group [5.02 (0.51) g/kg/day vs. 4.48 (0.17) g/kg/day, *p* = 0.001]. No significant differences were found in the lipid, carbohydrate, and energy intakes between the two arms (Table 2).

The same number of nutritional assessments were performed in both groups (mean 14, SD 4). Nutritional modifications (IQR) per patient were also similar (AF: 3 (2–4), TF: 4 (2–7); *p* = 0.165). Additional protein supplementation was provided at least once during the study to all patients (100%) in the AF group and to 21 out of 23 patients (91.3%) in the TF group. In the AF group, non-protein kilocalories were added for 40% of the patients.

### 3.2. Growth Outcomes

No significant differences were found in weight gain at 28 days after the initiation of fortification at 36 weeks of PMA, at discharge, or during the intervention (Table 3). No differences were observed in the difference in weight z score (SD) between the following different studied time points: at 28 days after initiation of fortification [AF −0.82 (0.62) vs. TF −1.18 (0.60), *p* = 0.09], at 36 weeks PMA [AF −1 (0.67) vs. TF −1.23 (0.75), *p* = 0.36], and at discharge [AF −0.9 (1.03) vs. TF −1.13 (0.57), *p* = 0.38].

There was no effect of the fortification type on length and HC growth, neither assessed in cm/week nor on changes in the z score (Table 4).

A multivariate analysis, incorporating the IUGR status and the randomization group, was performed to assess the impact on the response variables, with no significant effect of IUGR observed on weight velocity during the intervention (*p* = 0.99) or at any other evaluated time points.

### 3.3. Biochemical Parameters

Statistically significant differences were observed in urea at 36 weeks of PMA [AF 23 mg/dL (IQR 21–29 mg/dL) vs. TF 15 mg/dL (IQR 11.7–23 mg/dL); *p* = 0.026] and at the end of the intervention [AF 24 mg/dL (IQR 21–32 mg/dL) vs. TF 20 mg/dL (IQR 16–22 mg/dL); *p* = 0.018]. No differences were found in the phosphorus, calcium, or alkaline phosphatase values at any point.

**Figure 3:**
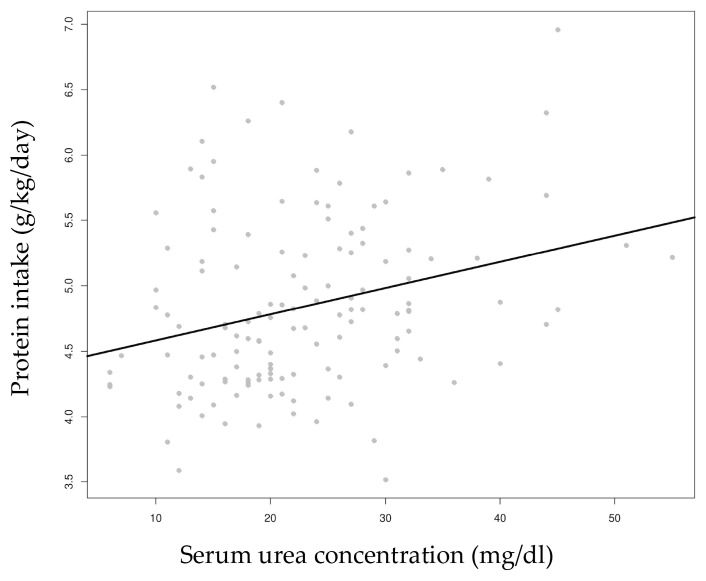
Correlation between protein intake and serum urea concentration.

A positive correlation was found between the protein intake and serum urea concentration (*p* = 0.002, Figure 3).

### 3.4. Neonatal Morbidity and Hospital Stay

No differences were found in the main neonatal outcomes, except for the presence of significant ductus arteriosus (AF group 47% vs. TF group 78%, *p* = 0.045). Late-onset sepsis was highly prevalent in both groups, with at least one episode of sepsis in 53% of patients in the AF group and 65% in the TF group (*p* = 0.46).

No differences were found in the NICU stay (SD) [59 (34) days in AF group vs. 58 (26) days in TF group, *p* = 0.915], hospital stay [84 (IQR 74–102) days in AF vs. 81 (IQR 74–102) days in TF, *p* = 0.79], and PMA at discharge [39.1 (IQR 38,4–40,6) weeks vs. 38.4 (IQR 37.9–40.3) weeks *p* = 0.40]. There was no difference in weight (SD) at the end of the intervention [2127 (481) g vs. 2120 (437) g, *p* = 0.96] nor at hospital discharge [2690.7 (589.4) g vs. 2816.1 (630) g, *p* = 0.542].

## 4. Discussion

The present study demonstrated no differences in postnatal weight gain and growth between the AF and TF groups across the different periods analyzed. To our knowledge, this study is unique with respect to increased energy intakes and the measurement of actual macronutrient intake in both groups. Our study took a rigorous approach to investigate the impact of individual fortification on growth of ELBW preterm infants.

While numerous studies have compared TF with SF, direct comparisons between AF and TF remain notably limited, and the existing studies suffer from considerable limitations.

Previous studies have demonstrated the superior growth outcomes of TF compared to SF [30,55,56,57]. A randomized clinical trial (RCT) comparing the three fortification methods found similar weight gain in the AF and TF groups, both of which were higher than in the SF group. In this study, protein intake was significantly higher in the individualized (AF and TF) groups compared to the SF group, with intakes of 4.3 g/kg/day in the AF group, 4.5 g/kg/day in the TF group, and 3.6 g/kg/day in the SF group [58]. However, it is important to note that only in the TF group was the actual composition of HM analyzed; in the other groups, the composition was estimated. Additionally, once the infants were randomized into their respective fortification groups, only protein fortification was increased, with no adjustments made to other macronutrients.

Rochow et al. [59] also compared SF with TF. Like our study, fortification was initiated with a standard fortifier, and fortification modules were added to adjust all HM macronutrients. However, the modules used for fat and protein fortification were different from ours and the control group received SF, which is inadequate for this population. They found greater weight at 36 weeks and greater growth velocity in the TF group.

Few studies have compared the AF method with TF. Bulut et al. [60] compared the effect of AF versus TF on growth, finding a positive effect in the TF group. However, the BUN limit used to increase protein intake was lower than in our study (5 mg/dL vs. 10 mg/dL) and their approach was limited, as they only supplemented protein in both groups. This restrictive strategy may not fully capture the potential benefits of a more comprehensive fortification regimen.

In contrast, our study is unique. Different from Arslanoglu et al. [26,27], who only increased protein intake, we increased caloric intake in the form of non-protein kilocalories for those patients with an inadequate growth response despite acceptable BUN concentration. This “modified” adjusted method, incorporating a more complete nutritional adjustment, facilitates a more appropriate comparison of the effects on growth between AF and TF. This thorough approach may also account for the absence of observed differences in growth between the two groups.

Protein intake is the primary growth factor when energy intake is appropriate [25,61]. The ESPGHAN recommends providing very preterm infants with 3.5–4.0 g/kg/day of protein. If growth remains slow, protein intake can be increased to up to 4.5 g/kg/day, as long as the protein quality is high and energy and other micronutrient intakes are adequate [25]. In our study, the protein intake in the TF group (4.48 g/kg/day) was within the recommended range [25], while the AF group exceeded these nutritional recommendations, with a protein intake of 5.02 g/kg/day. This “superfortification” was not linked to a corresponding rise in non-protein kilocalories, as no differences were observed in the energy, carbohydrate, and lipid intake. Therefore, as energy intake becomes a limiting factor when protein intake is high, it is likely that protein intakes >4.5 g/kg/day were not associated with a parallel increase in growth due to an inability to metabolize these proteins. Another explanation could be a potential ceiling effect for enteral protein supply, at least in the population studied, indicating that an enteral protein intake exceeding 4.5 g/kg/d might not further improve weight gain in this population. Our results are in line with data reported by Miller et al. [62], who also found no influence of increased enteral protein intake on weight gain in infants of similar gestational age at birth. In addition to adequate energy intake, when enteral protein intake is sufficient, the intake of other micronutrients can be a critical determinant of growth [25]. Indeed, it is important to consider that certain key micronutrients—such as choline, arachidonic acid, and docosahexaenoic acid, among others—are often present at levels significantly below fetal accretion in ELBW preterm infants, even in those fed fortified breast milk, and are not routinely supplemented. These micronutrients may play a pivotal role in the outcomes of these patients. However, any potential impact on the differences between the groups in our study is unlikely, as these micronutrients were not supplemented in any group.

Plasma urea, the product of amino acid oxidation, is considered an estimator of protein intake. A target urea level of 21–34 mg/dL or BUN 10–16 mg/dL has been proposed with limited evidence [1]. We found significantly higher urea levels in the high protein intake group and a positive correlation between the protein intake and plasma urea levels. This finding aligns with other authors who have supported the use of serum urea as an estimator of protein intake, although the appropriate levels still need to be defined [63].

Average growth rates during intervention were 16–17 g/kg/day, lower than previously reported with similar protein intakes but in the recommended ranges [25,59]. In our study, late-onset sepsis was not an exclusion criterion, possibly explaining lower weight gain. As growth is similar between both groups, it is not surprising that no differences are found in the main neonatal morbidities or the hospital stay.

On the other hand, we used modular products for enteral feeding available in our NICU, but evidence is scarce on their optimal composition for individual fortification.

Each method of fortification has several advantages and disadvantages. AF does not require an HM analyzer and urea determinations are part of routine blood tests in newborns. However, it requires frequent blood samples involving pain, discomfort, and the risk of anemia. AF may also involve a delay in nutritional adjustment as a metabolic response is needed [26,64].

TF has the advantage of adapting to changes in HM composition and adjusting different macronutrients to meet nutritional recommendations. The optimal frequency for testing HM per week to achieve an accurate intake has not been defined. Several studies have suggested that twice-weekly HM testing may provide adequate macronutrient intakes [33,65]. In addition, it requires the availability of trained staff in sample collection and maintenance and calibration of the HM analyzer. Therefore, it implies the need to consider material and human resources for its implementation. In any case, both fortification methods in our study required comparable nutritional modifications.

The strengths of the present study are the determination of actual macronutrient intakes with twice-weekly milk analyses in all infants and the length of intervention, which is larger than in previous studies. The analysis of macronutrient intake in both groups allows for a comprehensive evaluation of the impact of each type of fortification on the nutritional intake of preterm infants.

The study had certain limitations, especially the inability to stratify by IUGR. Nevertheless, a multivariate analysis was performed to assess the impact of IUGR on the response variables and no significant effect was observed. In addition, a minor error in the estimation of macronutrient intake was acknowledged, as the calculation was based on the composition of the predominant HM and limited to two days of measurement per week. These approaches allowed for the applicability of the study in case the results were positive. Additionally, the attending physician was not blinded and had access to both serum urea levels in the TF group and the nutritional prescriptions. However, this did not result in bias, as the research team retained sole responsibility for determining the nutritional strategy, thereby ensuring consistency and preventing deviations in the intervention.

## 5. Conclusions

Both AF and TF provide an adequate growth rate in ELBW infants. Therefore, the most feasible individualized fortification method should be chosen according to the resources available. At the protein levels provided by either fortification strategy, the impact of other critical nutrients on preterm infant outcomes should be considered. To verify effects on neurocognitive outcomes, a larger follow-up is needed.

## Figures and Tables

**Table 1:** General characteristics of study population.

General Characteristics of Study Population	Adjusted Fortification (n = 15)	Targeted Fortification (n = 23)	*p*-Value
**Gestational age (weeks)** Mean (95% CI)	27 (26.27, 28.01)	27 (25.88, 27.79)	0.64
**Birth weight (g)** Mean (95% CI)	765 (669.79, 860.74)	819 (767.77, 870.58)	0.26
**Birth weight z-score** Mean (95% CI)	−0.9 (−1.42, −0.38)	−0.3 (−0.79, 0.18)	0.09
**Length birth (cm)** Mean (95% CI)	32.5 (31.18, 33.82)	33.5 (32.51, 34.51)	0.19
**Length birth z-score** Median (95% CI)	−0.8 (−1.42, −0.24)	0.2 (−0.59, 0.97)	0.07
**Head circumference at birth (cm)** Mean (95% CI)	23.3 (22.42, 24.17)	23.6 (22.96, 24.16)	0.58
**Head circumference at birth z-score** Mean (95% CI)	−0.9 (−1.49, −0.36)	−0.45 (−0.98, 0.08)	0.21
**Male No (%)**	9/15 (60)	11/23 (47.8)	0.46
**Multiple gestation No (%)**	4/15 (26.7)	11/23 (47.8)	0.19
**Umbilical cord pH** Median (95% CI)	7.29 (7.17, 7.38)	7.33 (7.23, 7.36)	0.12
**Initial weight loss (%)** Mean (95% CI)	6.7 (4.30, 9.43)	6.18 (4.23, 8.12)	0.73
**Birth weight regain (day)** Median (95% CI)	6 (4.86, 7.81)	7 (5.34, 7.96)	0.47
**Weight at hospital discharge (g)** Mean (95% CI)	2691 (2364.26, 3017.07)	2816 (2543.68, 3088.49)	0.54
**Weight at hospital discharge z-score** Median (95% CI)	−1.9 (−2.5,−1.3)	−1 (−1.45, −0.59)	0.15
**Length at hospital discharge (cm)** Median (95% CI)	45 (43.57, 46.50)	45 (43.96, 46.29)	0.66
**Length at hospital discharge z-score** Median (95% CI)	−2.4 (−3.13, −1.71)	−2 (−2.32, −1.66)	0.32
**Head circumference at discharge (cm)** Mean (95% CI)	33.7 (32.85, 34.45)	33.2 (32.52, 33.96)	0.43
**Head circumference at hospital discharge z-score** Mean (95% CI)	−0.85 (−1.5, −0.20)	−0.9 (−1.35, −0.46)	0.89

CI: confidence interval.

**Table 2:** Macronutrient analysis of human milk (HM) and actual macronutrient intake in adjusted fortification group and targeted fortification group.

HM Analyses	Adjusted Fortification (n = 15)	Targeted Fortification (n = 23)	*p*-Value	Intake	Adjusted Fortification (n = 15)	Targeted Fortification (n = 23)	*p*-Value
**Protein HM (g/100 mL)** Mean (95% CI)	1.34 (1.25, 1.45)	1.27 (1.21, 1.35)	0.20	**Protein g/kg/day** Mean (95% CI)	**5.02 (4.73, 5.30)**	**4.48 (4.41, 4.56)**	**0.001**
**Lipid HM (g/100 mL)** Median (95% CI)	3.30 (3.16, 3.90)	3.20 (3.05, 3.34)	0.10	**Lipid g/kg/day** Mean (95% CI)	7.15 (6.45, 7.85)	6.56 (6.39, 6.74)	0.10
**Carbohydrates HM (g/100 mL)** Median (95% CI)	7.32 (7.23, 7.77)	7.61 (7.35, 7.79)	0.80	**Carbohydrates g/kg/day** Mean (95% CI)	15.15 (14.09, 16.22)	15.15 (14.54, 15.76)	0.99
**Energy HM** **(kcal/100 mL)** Median (95% CI)	65.66 (63.67, 69.96)	64.08 (62.78, 65.57)	0.15	**Energy kcal/kg/day** Mean (95% CI)	144.72 (135.62, 153.83)	137.63 (134.94, 140.32)	0.13

CI: confidence interval.

**Table 3:** Weight gain (g/kg/day) in adjusted fortification group and targeted fortification group.

Weight Gain g/kg/day	Adjusted Fortification (n = 15)	Targeted Fortification (n = 23)	Difference (95% CI)	*p*-Value
**From birth to 28 days from the start of HM fortification ** Mean (95% CI)	14.06 (12.31, 15.81)	13.24 (12.13, 14.36)	0.82 (−1.19, 2.83)	0.38
**From birth to 36 weeks PMA** Median (95% CI)	15.31 (13.37, 16.42)	14.02 (13.16, 16.41)	0.64 (−1.17, 2.33)	0.53
**From birth to discharge ** Median (95% CI)	14.53 (13.01, 15.46)	14.04 (13.35, 15.12)	0.37 (−1.47, 1.52)	0.68
**During the intervention ** Mean (95% CI)	16.33 (15.26, 17.40)	16.89 (15.44, 18.34)	−0.56 (−2.30, 1.18)	0.56

PMA: Postmenstrual age. CI: confidence interval.

**Table 4:** Length and head circumference growth in adjusted fortification group and targeted fortification group.

Length Growth cm/week	Adjusted Fortification (n = 15)	Targeted Fortification (n = 23)	Difference (95% CI)	*p* Value	Head Circumference Growth cm/week	Adjusted Fortification (n = 15)	Targeted Fortification (n = 23)	Difference (95% CI)	*p* Value
**From birth to 28 days from the start of HM fortification ** Mean (CI 95%)	0.91 (0.78, 1.05)	0.86 (0.74, 0.98)	0.05 (−0.12, 0.23)	0.536	**From birth to 28 days from the start of HM** **fortification** Mean (CI 95%)	0.78 (0.65, 0.93)	0.73 (0.64, 0.83)	0.06 (−0.10, 0.22)	0.44
**From birth to 36 weeks PMA ** Median (CI 95%)	0.95 (0.89, 1.08)	0.88 (0.70, 1.49)	0.07 (−0.12, 0.20)	0.38	**From birth to 36 weeks PMA ** Median (CI 95%)	0.85 (0.76, 0.94)	0.86 (0.70, 1.18)	0.01 (−0.10, 0.13)	0.86
**From birth to discharge ** Median (CI 95%)	0.91 (0.89, 1.07)	0.94 (0.79, 1.33)	0.04 (−0.07, 0.15)	0.47	**From birth to discharge ** Median (CI 95%)	0.83 (0.75, 0.91)	0.79 (0.67, 1.09)	0.03 (−0.06, 0.13)	0.41

PMA: Postmenstrual age. CI: confidence interval.

## Data Availability

The datasets generated during and/or analyzed during the current study are available from the corresponding author on reasonable request. Researchers submitting a methodologically sound proposal should contact miguel.saenz@salud.madrid.org. In order to access, requesters will need to sign a data access agreement.

## Supplementary Materials

The following supporting information can be downloaded at: https://www.mdpi.com/article/10.3390/nu16172904/s1, Figure S1. Table to calculate the actual intake of macronutrients after fortification. The patient’s weight, total volume of human milk and the composition per 100 mL of macronutrients resulting from milk analysis should be filled in. Figure S2. Example of nutritional adjustment in a targeted fortification group patient.
