# Supplementary material for: Adjusted versus Targeted Fortification in Extremely Low Birth Weight Preterm Infants: Fortin Study—A Randomized Clinical Trial
Source: Nutrients. 2024 Aug 30;16(17):2904. doi: 10.3390/nu16172904 (PMC11397412; doi:10.3390/nu16172904)
Supplement: Supplementary file 1 [file nutrients-16-02904-s001.zip › nutrients-3150382-supplementary.pdf]

Supplementary Materials: Targeted fortification.

**Patient:**							**Patient weight (g)**
	**Yes=1; No=0**	**Intake volume (ml)**	**Protein**	**Carbohydrates**	**Lipids**	**Energy Kcal**	
Human milk	-		0	0	0	0	
FM85 4%	0	0	0	0	0	0	
DMT 2%	0	0	0	0	0	0	
DMT 3%	0	0	0	0	0	0	
DMT 4%	0	0	0	0	0	0	
MCT 1 ml	0	0	0	0	0	0	
MCT 1,5 ml	0	0	0	0	0	0	
MCT 2 ml	0	0	0	0	0	0	
MCT 3 ml	0	0	0	0	0	0	
Olig. 0,5g/100ml	0	0	0	0	0	0	
Olig. 1g/100ml	0	0	0	0	0	0	
Olig. 1,5g/100ml	0	0	0	0	0	0	
Total	0		0	0	0	0	
Total/kg							
Rec./kg/day	135-200		3.5-4.5	11.6-13.2	4.8-6.6	110-135	

	**Human milk composition per 100ml**	**Protein**	**Carbohydrates**	**Lipids**	**Energy**
Human milk	100 ml.				

Figure S1. Table to calculate the actual intake of macronutrients after fortification. The patient's weight, total volume of human milk and the composition per 100ml of macronutrients resulting from milk analysis should be filled in.

**Patient:**	3						**Patient weight (g)**
	**Yes=1; No=0**	**Intake volume (ml)**	**Protein**	**Carbohydrates**	**Lipids**	**Energy Kcal**	1180
Human milk	-	184	2,0792	13,432	6,0904	116,84	
FM85 4%	1	7,36	2,6128	2,38464	1,33216	32,016	
DMT 2%	0	0	0	0	0	0	
DMT 3%	0	0	0	0	0	0	
DMT 4%	0	0	0	0	0	0	
MCT 1 ml	0	0	0	0	0	0	
MCT 1,5 ml	0	0	0	0	0	0	
MCT 2 ml	0	0	0	0	0	0	
MCT 3 ml	0	0	0	0	0	0	
Olig. 0,5g/100ml	1	0,736	0,59616	0,02208	0,00736	2,5392	
Olig. 1g/100ml	0	0	0	0	0	0	
Olig. 1,5g/100ml	0	0	0	0	0	0	
Total	184		5,28816	15,83872	7,42992	151,3952	
Total/kg	155,9322034		4,481491525	13,42264407	6,296542373	128,3010169	
Rec./kg/day	135-200		3.5-4.5	11.6-13.2	4.8-6.6	110-135	

	**Human milk composition per 100ml**	**Protein**	**Carbohydrates**	**Lipids**	**Energy**
Human milk	100 ml.	1,13	7,3	3,31	63,5

Figure S2. Example of nutritional adjustment in a targeted fortification group patient.
